# An Education and Support Chatbot for Postpartum Mothers With Opioid Use Disorder: Qualitative Needs Assessment

**DOI:** 10.2196/96237

**Published:** 2026-10-21

**Authors:** Emma Farber, Mlka Mengesha, Erica Reisinger, Nicole Ellis, Andrew Lu, Peter F Cronholm, Nia Bhadra-Heintz, Kirstin Leitner, Navid Roder, Sara L Kornfield

**Affiliations:** 1 Center for Women's Behavioral Wellness, Department of Psychiatry, University of Pennsylvania, Perelman School of Medicine, 3535 Market Street, 3rd floor, Philadelphia, PA, 19104, United States; 2 Mixed Methods Research Lab, Department of Family Medicine and Community Health, University of Pennsylvania, Perelman School of Medicine, Philadelphia, PA, United States; 3 Department of Psychiatry, University of Pennsylvania, Perelman School of Medicine, Philadelphia, PA, United States; 4 Center for Addiction Medicine & Policy, Department of Emergency Medicine, University of Pennsylvania, Perelman School of Medicine, Philadelphia, PA, United States; 5 Leonard Davis Institute of Health Economics, Penn Implementation Science Center, Center for Clinical Epidemiology and Biostatistics, Center for Public Health, University of Pennsylvania, Perelman School of Medicine, Philadelphia, PA, United States; 6 Department of Family Medicine and Community Health, University of Pennsylvania, Perelman School of Medicine, Philadelphia, PA, United States; 7 Department of Obstetrics and Gynecology, University of Pennsylvania, Perelman School of Medicine, Philadelphia, PA, United States

**Keywords:** chatbot, needs assessment, OUD, postpartum, maternal health, substance abuse recovery, opioid use disorder

## Abstract

**Background:**

The prevalence of opioid use disorder (OUD) among women giving birth in US hospitals continues to rise. Increasing fatalities due to perinatal drug overdose have in part been attributed to decreased access to social support and treatment services. In recent years, mobile health (mHealth) technologies have been used to provide care to patients outside of traditional clinical settings. Given that perinatal OUD treatment works best when there is frequent contact between patient and clinician, harnessing mHealth could be valuable for improving patient engagement in perinatal OUD care.

**Objective:**

To address the increasing epidemic of perinatal OUD, which is compounded by loneliness and social isolation, this study aimed to adapt an existing mHealth chatbot intervention for postpartum individuals by developing educational and supportive content relevant to people in the postpartum period experiencing OUD and recovery.

**Methods:**

We used a participatory design to conduct a needs assessment via in-depth qualitative interviews with patients (n=12) and providers (n=11). Interview data were analyzed using NVivo software (Lumivero) according to a priori codebooks and using inductive coding, which identified major themes to address in a future adaptation of an interactive chatbot. An advisory board was convened to review the data and contribute to content generation that would be most useful for perinatal mothers who are in treatment for OUD.

**Results:**

Patients and providers agreed that an interactive chatbot would be useful for patients seeking information on childbirth recovery, substance use recovery, pain management, and infant care, including feeding. Patients specifically wanted a chatbot that felt personal and addressed their individual and specific needs. Providers indicated that the chatbot should address topics that came up frequently in after-hours phone calls to reduce provider burden.

**Conclusions:**

Conducting an in-depth participatory-driven needs assessment will support the refinement of an existing chatbot to better meet the needs of a high-risk, difficult-to-reach population. As we move forward to test the chatbot for acceptability and feasibility, data from the needs assessment will support ongoing iterative enhancement of the content, delivery, and safety of the chatbot.

## Introduction

### Background

The US opioid crisis has had a significant effect on women of reproductive age both nationally and regionally [1,2]. Increasing numbers of women report opioid misuse during pregnancy; overall, of women who were surveyed as part of the Pregnancy Risk Assessment Monitoring Study [3], 21% of those using prescription opioids during pregnancy reported misuse, and 36% of those reported wanting or needing to cut back their use [4].

The postpartum period can heighten the risks of adverse outcomes and overdose among women struggling with opioid use disorder (OUD) [5]. Reasons for this include exacerbation of psychiatric comorbidities, increased experiences of partner violence, infant hospitalizations for Neonatal Opioid Withdrawal Syndrome (NOWS), as well as possible engagement with child welfare systems related to the substance use [5-8]. Mounting evidence has shown that women are more likely than men to face barriers to treatment entry and access, have a faster-growing rate of hospitalizations due to substance use, and a faster increase in the rate of overdoses, all of which point to the need for interventions specific to this population [9-11].

Loneliness and social isolation are compounded during the perinatal period and contribute to OUD and treatment outcomes [12]. Specifically, both loneliness and social isolation have been linked to increased rates of use, amount, relapse, and overdose, even when controlling for medications for opioid use disorder (MOUD) treatment engagement, employment, relationship status, and mental health diagnoses [13-15]. Compellingly, the model of social homeostasis suggests that opioid drugs in particular target neurobiological circuits implicated in social bonding, which have been posited to increase vulnerability to opioid misuse or addiction in the absence of meaningful social connection [16]. Feeling connected to others is especially critical during postpartum periods as loneliness is associated with postpartum depression [17,18] and poor social support can impact treatment outcomes [19].

### Prior Work

Recent research highlighting the effect of maternal loneliness on worsening mental health outcomes has suggested that the use of text messages for support can be a more useful intervention than engagement with social media or online forums [20]. Beyond providing patients with easy-to-access information and a sense of connectedness, mobile health (mHealth) can also reduce provider burden in caring for a high-need population [21]. While access concerns to the internet and smart devices have been raised, this is generally not a problem among racially diverse and lower-income populations [22].

Educational content delivered asynchronously and “in real words” may be a way to overcome provider burden and simultaneously address patients’ concerns and questions. Indeed, mHealth technologies, such as apps, chatbots, and SMS messaging, allowing texting and feedback from digital support mechanisms to provide clinical education and support in a relational context, have been shown to be acceptable to perinatal patients with a range of clinical needs [23,24].

The limited mHealth technologies that exist for the support of perinatal OUD indicate high acceptability and usability. Digital delivery of education on NOWS, infant care, postpartum care, and maternal addiction recovery has the potential to empower a historically stigmatized patient population and support treatment adherence by increasing access to clinical support [25-27]. While these studies demonstrate that mHealth can be a valuable communication and education tool for perinatal patients with OUD, there are no studies that explore the acceptability of using an interactive text-based chatbot for these purposes.

Current technology models for perinatal patients with OUD cite the importance of patient-centered design [25,27]. Rooted in user-centered design, an approach to product development by focusing on target users [28,29], participatory design engages the intended users as stakeholders in a collaborative research process to ensure patient needs are effectively met [30]. Qualitative feedback from patients with OUD and health care providers can be used to facilitate the development of equitable, high-quality mHealth technologies to sensitively support postpartum individuals, promote addiction care, and address mental health disparities [31,32]. There is high utility in conducting a needs assessment to identify barriers, facilitators, and the specific needs of the target population to develop the most useful and acceptable intervention [33]. Therefore, qualitative feedback is critical to the development of text message content and question-and-response information for a postpartum OUD support chatbot that reflects the topics of importance, resources of interest, and preferred wording, tone, and language of its intended population.

### Study Objectives

To this end, we conducted an in-depth needs assessment with postpartum individuals with OUD and health care providers engaged in the treatment of perinatal patients with substance use concerns to adapt our team’s previously developed “Healing at Home” for postpartum support [34], a 24/7 2-way messaging chatbot, to the needs of a perinatal population with OUD. We will call this adapted intervention the “Penny Chatbot.” Penny will be a rule-based chatbot that sends anticipatory guidance, responds to keywords, and follows “if/then” decision tree logic.

## Methods

### Study Design

We conducted a needs assessment using semistructured interviews to gather data on the needs and preferences of perinatal patients with OUD and the clinicians who care for them. We shared this information with a stakeholder advisory board composed of health care providers engaged in OUD care (including clinicians, social workers, and certified recovery specialists) and parent participants with lived experience of OUD to develop educational and supportive content relevant to OUD and recovery in the postpartum period. This work was guided by the COREQ (Consolidated Criteria for Reporting Qualitative Research) guidelines [35].

### Participants

Two stakeholder groups were recruited to take part in 30‐60 minute semistructured interviews as part of the assessment. The mother sample included patients from the Perinatal Resources for Opioid Use Disorder (PROUD) clinic who were biological mothers who had given birth to a live infant within the last 2 years. Potential patients were referred by clinicians and approached by the study team via phone call or text message. The provider sample included certified peer recovery specialists, social workers, and clinicians from the departments of Obstetrics and Gynecology, Family Medicine, Addiction Psychiatry, Emergency Medicine, and Psychiatry. Provider participants all had extensive experience treating or working with perinatal patients with OUD, which made their expertise valuable for chatbot development. Providers are aware of the most commonly asked questions, important education relevant to postpartum OUD, and the facilitators and barriers to mHealth and treatment delivery for the target patient population. We determined that approximately 12 patients per group would be sufficient to reach thematic saturation and identify issues that would be useful for content development [36]. Semistructured interviews were conducted with 12 perinatal participants with OUD and 11 providers.

### Data Collection Procedures

Patient interviews were conducted in-person and clinician interviews were conducted via Zoom (Zoom Communications, Inc). All interviews were conducted individually, audio-recorded with participants’ permission, deidentified, and then transcribed by a professional transcription service. Interviews were conducted by a female senior research coordinator who has extensive experience collaborating with perinatal patients with OUD, both in her role as a researcher and due to her cross-training as a birth and postpartum doula. She acknowledged that patients’ willingness to openly disclose their opinions and experiences with perinatal OUD may be hindered by a power imbalance, fear of stigma, or social desirability. Prior to initiating the interview, she took time to build rapport with the patient participants. She assured patients that their responses were anonymous and there were no right or wrong answers. She added that their expertise was important and the research team wants to better understand how to support perinatal individuals with OUD.

Separate interview guides were developed and used for each study group to collect both perspectives and reflect a holistic understanding of stakeholders’ perspectives and values to inform chatbot content. The interview guides were developed using input from stakeholders with lived experiences (ie, certified recovery specialists [CRSs]) and providers who routinely work with the patient population. Patient interviews included questions to gather data on patient-oriented content, such as “What questions do you have about managing your OUD care during your pregnancy?” We also posed questions related to delivery method and types of messages sent, including “How often would you like information tailored to your gestational age or baby’s age ‘pushed’ to you as a text message?” and “How do you see yourself using a texting option to get information about your healthcare, your baby’s development, or your recovery?”

Questions for clinicians and CRSs focused on commonly asked patient questions, for example, “What are some of the most frequently asked questions from patients about managing OUD in the postpartum?” The interview also probed for ways in which clinicians have successfully or unsuccessfully engaged patients in other telehealth platforms and anticipated patient reactions to a text-support platform, including “Do you use messaging systems to stay in touch with your patients between visits?” “How do you think your postpartum patients with OUD would respond to a texting platform to support them and help answer questions for them?”

### Qualitative Analysis

Codebooks were developed using an integrated approach [37]. This process was informed by modified grounded theory [38], combining inductive coding of emerging themes within the data along with deductive codes informed by a priori determined attributes of interest [39] (ie, specific perinatal and OUD treatment information, and messaging information).

Qualitative analysis was conducted by staff at the Mixed Methods Research Lab (MMRL) at the University of Pennsylvania in a manner consistent with institutional review boards (IRBs) and federal Health Insurance Portability and Accountability Act (HIPAA) guidelines and regulations. One of the MMRL coders is a master’s level statistician with a background in public health, maternal mental health, and psychology who possesses more than 4 years of experience. The other coder is a BA-level trainee under the supervision of the master’s level statistician and has a background in psychology and statistical analysis. They both undertook extensive training in qualitative research methodologies and software packages when they were onboarded. MMRL faculty and staff are highly trained and expert in managing, conducting, and analyzing protocol designs involving grounded theory and other methodologies. Scientific oversight for this study was provided by MMRL director and coauthor PFC, MD, MSCE, who has more than 20 years of qualitative and mixed methods experience, and other MMRL faculty and senior researchers.

Using NVivo software (version 15; Lumivero), 2 coders from MMRL independently conducted open coding of 2 to 3 transcripts per study group, identifying initial themes and patterns in the data. Preliminary codebooks were developed through meetings in which coders compared coding and discussed interpretations. The codebooks were iteratively refined during coding and interrater reliability (IRR) tests to ensure clarity and consistency. For the IRR tests, codes were applied to approximately 25% of the dataset per group (patient n=3; provider n=3), double-coded independently by both coders. Coders then met through multiple sessions to review and discuss discrepancies and reach consensus until strong agreement was reached for all codes (Cohen κ>0.75). Following this process, the remaining data were coded independently by the primary coder (MM) using the finalized codebooks, cross-referencing identified thematic categories against the raw transcripts to ensure consistency between the data and our findings. Analyses progressed as codes were then grouped into broader themes and constructs for each group. Themes were also analyzed across participant groups to identify patterns, noting any overlap or variation in similar topics.

### Ethical Considerations

This study was reviewed by the University of Pennsylvania IRB (protocol number 853729). According to 45 CFR 46.104, category 3, 2, the proposal met eligibility criteria for IRB review exemption. Pregnant patients were recruited to participate in the study. To protect a potentially vulnerable group from undue influence or coercion, all patient participants were required to be aged 18 years or older, able to consent on their own, and have no contraindications for participation. If there was any reason during the informed consent process that led to suspicion that the participant’s mental state was impaired enough to cause doubt about the validity of the consent statement, they were excluded. Participants were informed that participation is completely voluntary and they may opt out at any time. Those who chose to participate provided consent electronically through REDCap (Vanderbilt University) in accordance with the IRB policies on collecting electronic consent. Data were anonymized and deidentified. Participants were compensated US $20 on a Greenphire ClinCard for their participation in the one-time interview.

## Results

### Participant Demographics

Table 1 reports the demographics of the sample. Patients were 50% White, the majority were single, and most had low incomes. Half the sample were in current treatment for OUD at the time of participation and half were currently pregnant. All patients had given birth to a child within the last 2 years, although child age data were not available.

**Table 1.** Participant demographic characteristics.

Characteristics	Patients (n=12)	Providers (n=11)
Age (years), mean (SD)	33.5	40.2
Race, n (%)		
White	6 (50)	8 (73)
Ethnicity, n (%)		
Hispanic	2 (17)	0 (0)
Non-Hispanic	10 (83)	11 (100)
Patient household income (US $), n (%)		
<$25,000	11 (92)	—^a^
Patient marital status, n (%)		
Single	8 (67)	—
Patients currently pregnant, n (%)	6 (50)	—
Patients using medication for OUD^b^ and psychotherapy, n (%)	6 (50)	—
Patients only using medication for OUD, n (%)	5 (42)	—
Patients not in treatment, n (%)	1 (8)	—

a Not available.

b OUD: opioid use disorder.

### Patient Perspectives

#### Overview

Six themes emerged from the qualitative data analysis of the patient interviews. These included childbirth and postpartum recovery experiences, substance use and withdrawal, infant feeding practices, finding support and information, communication preferences, and acceptability of the proposed Penny Chatbot.

#### Theme 1: Postbirth Pain and Mental Health

Patients began interviews by discussing their most recent pregnancy and childbirth experiences, during a time that aligned with their substance use and/or OUD treatment. Childbirth and postpartum experiences that were described related to managing physical pain and mental health concerns.

*Because I had sickle cell, so I took narcotic medicine regardless if I took the Suboxone or not. So, when you ... are in chronic pain and stuff, you take that kind of medicine. Then, I thought that the Suboxone medicine didn’t really work, but at first, I didn’t really give it a try, but then once I got pregnant, I said, “I got to take something. I can’t take that medicine all the time*.

[Patient 104]

*But I definitely foresee myself for labor, like I don’t want opioids. I don’t want any opiates during labor or after labor or anything. So how that changes or how that might change postpartum recovery.*

[Patient 112]

Mental health concerns were also brought up by several patients. Concerns about perinatal depression and anxiety were most common, with patients questioning the safety of psychiatric medication use.

*I have like, anxiety, depression and all that stuff. You know what I mean? Like, I probably felt myself maybe getting a little bit depressed and unmotivated to do things... And that was because I was back and forth with all my psych meds. I started taking them again when I was pregnant. And then I found out the Wellbutrin and all that stuff that I was taking can kind of be potentially risk to the baby. So, I stopped taking it and I was like, I’ll just take it if I need it.*

[Patient 109]

#### Theme 2: Substance Use and Withdrawal

Patients discussed their concerns about their opioid use and the medications used to manage recovery. Themes that emerged centered around patients’ concerns with the potential effects on fetal and child development, and the baby’s potential for experiencing withdrawal after birth.

*I said, “Okay. Does the medicine go straight to him? Does he feel any of this?” and they were telling me that he might get withdrawal and I said, “Okay. If he does get withdrawal, what does that look like? What does he go through when he gets withdrawals that when it gets to a point where I have to bring him to the hospital...*

[Patient 101]

*So, I want to know how bad would the withdrawal be with my baby and with me. I don’t want my baby to have to...I’m not worried about me...I just don’t want my baby to have to go through that.*

[Patient 104]

*I had questions about how long she would be irritable and in pain after the medicine. And I had a lot of questions about that.*

[Patient 110]

*...Withdrawal in me with the baby that was a concern, but I know that’s gonna happen, I already know that... I think I’m doing pretty good... but in the beginning I was dipping that way, so I was a little concerned about that...But I’m not sure how that affects the baby in the beginning...*

[Patient 111]

#### Theme 3: Infant Feeding Practices

Patients were asked if they had questions for their providers regarding various topics, such as their personal health during pregnancy and postpartum, the health and care of their newborn, and their recovery from both childbirth and OUD.

Questions about feeding, and more specifically, the safety of breastfeeding while using medications for OUD, were frequently mentioned:

I asked, why isn’t he latching? I didn’t understand. Why he barely didn’t eat.

[Later in the interview] ...*if I took a higher dose than I was supposed to be because of extra craving and I’m breastfeeding- then, I think I would sit there and ask, “Hey, would it be okay if I do this?” and then breastfeed her after that.*

[Patient 101]

*My questions was is the baby going to have withdrawal from Suboxone?’, and then, they said if I breastfeed, then that would make the withdrawal not as bad. So, I said, ‘Okay. I will breastfeed the baby a little bit.*

[Patient 104]

*The only concern that I had at first was like they tell you it’s okay to breastfeed, but I just can’t understand how it’s okay for you to breastfeed knowing you’re on Suboxone? It doesn’t make sense to me.*

[Patient 108]

Patients who were formula feeding also indicated that using fortified formula might be challenging, given that it is prepared differently than typical baby formula.

*Like they gotta show me how to mix up his formula because it’s like fortified with different, you know, with like a picture and stuff.*

[Patient 109]

#### Theme 4: Finding Support and Information

Patients discussed sources of support in their lives, from their medical team to family and friends. All patients expressed satisfaction with the care and support they received at their OUD care facility. Many patients reported receiving both emotional and instrumental support from loved ones, including their partners, parents, other family members, friends, children, and members of their religious communities.

There were mixed reports on the level of comfort patients felt in confiding in sources of support when experiencing difficulties with recovery, unless the other parties had personal experience with OUD. Patients expressed that finding support from other postpartum individuals in OUD recovery was ideal. This was particularly impactful because it enabled connection through shared experiences.

*My support system was somebody that introduced me to the program, which is also a mom. She’s my best friend...She’s in the program when she has children. So, that was my support person most of the time because she was just basically, keep me on the straight and narrow line like you got this.*

[Patient 110]

Several patients reported using the internet as an additional source of information and support, with varying degrees of success or satisfaction. These sources included online search engines, online support groups, and forums.

*I ask Google everything.*

[Patient 104]

*And I didn’t bring it up to the doctors. I didn’t — I Googled it and, yeah, that was about it. I didn’t really get an answer to it.” [Later in the interview] ... “Is my daughter supposed to be talking at two years old, is she supposed to be pretending, like, just like different things. I Google everything. But, yeah, sometimes the answer is not right.*

[Patient 110]

*I would go on to an online forum and talk to people there or read through forums there. Like I said on Reddit relieves, there’s a lot of recovery communities and it’s so much easier sometimes to just post anonymously, everything that you wanna get out and then to get messages back from strangers. Like, oh, I’ve gone through the same thing da da da da. And I think that’s really been a really helpful resource for me in all the years I’ve been in and out of recovery.*

[Patient 112]

#### Theme 5: Communication Preferences

Patients were asked about their preferred methods of communication with their providers and with a hypothetical OUD recovery support program. Responses indicated that preferences for text messaging, phone calls, and in-person conversations depended on the context of the conversation or the nature of questions patients had. Discussions about texting highlighted its convenience and effectiveness when a phone call was not possible or preferred, or when seeking answers to general questions.

*Sometimes, I don’t want to talk. I rather text...because like you said, sometimes [things come up] at like 4:00 in the morning.*

[Patient 105]

*... text messaging is definitely a good thing because some people don’t like talking on the phone, but then there are people who would rather speak to somebody. You know, like a suicide hotline. They just need that reassurance, that soft/warm voice, or...just letting you know that it’s going to be okay. If you guys could do something like that as well, like, “Okay, they have the option of having a text message” or having the option of ... a peer specialist doing calls for moms who are going through this. Moms that are going through this, they would rather text because they’ve been there.*

[Patient 106]

*Like finding out a diaper rash cream that’s like the best, or the best Pampers or best Eczema soap, things like that. Where I had something, somebody I could just text versus go to Google articles for hours, I think that would have been really helpful.*

[Patient 110]

*I feel like it would probably be easier. I hate having to call places and deal with things on the phone even if it’s just a doctor’s office. It’s always easier for me like email or to send a doctor a message or anything like that. So, I think the chat option sounds like a really good tool.*

[Patient 112]

Some patients explained that Penny could reduce the frequency with which they search for answers online or call their doctor’s office, especially if responses included explanations and resources rather than simple answers that may cause further confusion.

*I definitely think this [chatbot] is a really good program because a lot of moms and dads run straight to Google. So, for this to be able to like answer certain questions in regards to the development of the kids and this program- this is really a good program.*

[Patient 110]

*If it were to direct me to other resources or provide more than just a, oh yes, this is bad or no, this is not bad. Like I said, just having the science behind why or the actual explanations behind why these things might be good or bad and being able to make my own choice about it based off of that...So a mixture of what your actual doctor would say and what it means...for the baby, for me.*

[Patient 112]

Opinions around appropriate frequency of messaging were mixed. Most patients indicated that they would prefer messages more frequently (ie, from daily to at least weekly). Some respondents shared that they would want messages less frequently as they might be overwhelmed by them. One patient noted that more frequent messaging may be useful in the early postpartum stages.

A common theme emphasized by patients was that they would not like messages that felt automated or impersonal. Patients did not want to feel like they were receiving the same messages and responses as others.

*I feel like I’m gonna go back to it, being personalized. A number one thing for that and I feel like the program could be a huge success if you guys do individual, like if you're able to, you know, I know it might be a little bit hard in the beginning, but to individualize someone else’s needs and stuff like that is definitely a plus.*

[Patient 106]

Most patients reported that they would enjoy receiving encouraging texts about their recovery. Because the postpartum period can be a vulnerable and sensitive time for patients, one individual expressed the importance of messaging that felt personalized and acknowledged common obstacles this group might be facing.

#### Theme 6: Acceptability of the Proposed Penny Chatbot

Patients reported frequently using online sources to get answers to their questions or concerns. Because of this, almost all patients agreed that they would like to use a text messaging-based system such as Penny. Patients noted that they would use the Penny Chatbot to inquire about OUD medication use and its impact on breastfeeding, personal care, best products to use during postpartum recovery, concerns surrounding a child’s health or development, support, and information on relevant resources. Patients expressed interest in Penny because it would be an option available at all times, is more convenient than calling their doctor’s office, and is a more trusted and accurate option when compared to searching for answers to questions online.

### Clinicians’ Perspectives

#### Overview

Three major themes emerged from the analysis of clinician responses. They included perspectives and experiences with telehealth, commonly asked questions for providers, and perspectives on the use of Penny.

#### Theme 1: Telehealth and Remote Monitoring

Clinicians discussed their opinions and experiences with telehealth platforms to provide clinical care. Almost all clinicians described using this mode of care very frequently. Many clinicians described familiarity with telehealth or remote monitoring platforms, such as telehealth video visits and a postpartum high blood pressure monitoring program called Heart Safe Motherhood [40], that uses similar text messaging methods as our proposed intervention. Clinicians also described using other forms of communication to connect with patients, such as phone calls or text messaging during the postpartum period.

Clinicians expanded on their experiences with telehealth and discussed the perceived benefits and limitations of this mode of care. Although an effective and, at times, more ideal option than in-person care, clinicians agreed that a mix of both modes of care is the most ideal scenario for much of this patient population. Clinicians also noted that this may change depending on the patient and their individual needs. Due to the specialized health and needs of the study population, these patients may require direct, frequent in-person care. Even so, incorporating telehealth was viewed as being able to help patients stay engaged with their care if needs or issues arise that would impact or prevent them from being able to come into the office.

Clinicians shared their perceptions of the barriers to patients using telehealth effectively. These include unreliable phone service or internet connection and competing priorities or concerns, both of which are extremely common in this patient population. One clinician described that telehealth options can contribute to gaps in care. Specific to our population of interest, clinicians noted that treatment plans and care may require in-person testing to ensure medication adherence, which cannot be conducted through telehealth. The ease of telehealth may also deter patients from seeking in-person care when it may be necessary, such as if physical testing is required. Another challenge providers reported with telehealth is that it may be more difficult to establish rapport with patients as compared to in-person care.

*Sometimes just the human connection. I know that one of our substance-use navigators sees patients in person in the [clinic blinded for review] and she is definitely involved in their lives. Because she made that connect like it’s an in-person relationship. You know, she’s been in the labor and delivery room and she’s been to their homes or in the community. So, I don't think it’s a replacement but I do think that the relationship cultivates differently.*

[Provider 101]

Clinicians noted that telehealth is ideal in situations where concerns can be addressed quickly and between in-person visits to ensure follow-up and adherence to a treatment plan or engagement in health care. This option is essential when patients have barriers to attending in-person appointments, such as transportation, scheduling, or childcare issues. Telehealth options ensure that patients do not experience gaps in care if they cannot attend an in-person visit. One provider noted that telehealth may also be ideal for addressing concerns that cannot be addressed within in-person visits due to appointment time constraints. Overall, clinicians described feeling that telehealth promotes engagement in care and adherence to treatment as patients receive more contact with clinicians than via traditional in-person-only care.

When working with this more vulnerable population, clinicians explained that being available to patients through phone calls or text messages may improve and strengthen the provider-patient relationship. This increased access to providers can make patients feel more trust toward and support from clinicians, which may encourage engagement and adherence to treatment and care. Some issues expressed by clinicians about being more accessible to their patients included misaligned patient expectations and needing to establish boundaries for this type of contact. Clinicians shared instances in which patients would initiate contact outside of business hours and unrealistic expectations for provider response time.

*I think also it does sometimes create a sense—if that’s the word, not urgency but I think when somebody sends a text message to us, they're expecting an immediate response. I can tell you right before this meeting, I got a text message from somebody that was like, "Hey, can I have transportation assistance for my ultrasound tomorrow?" I didn't respond for 10 minutes and I already got a bunch of question marks...*

[Provider 102]

#### Theme 2: Questions for Clinicians

Clinicians were asked to share the most frequently asked questions or concerns that they receive from patients. When managing OUD in the postpartum period, most patients raise concerns and questions about breastfeeding safety while using MOUDs, medication management, recovery, withdrawal (for patient and infant), resources such as transportation or infant-specific needs, and general postpartum and infant care.

Mental health and emotional support are topics that patients may not typically inquire about, but are prevalent and important to monitor among this patient population. Clinicians discussed the comorbidity of internalizing disorders, such as anxiety and depression, with OUD. Providing education to patients on mental health risk is frequently prioritized.

*So to me it’s like, I don't spend so much time on things that are opioid use disorder specific and I also talk a little bit about sometimes there’s a lot of overlap between…patients that have opioid use disorder and also have behavioral health diagnoses, have a lot of anxiety or kind of prone to depression to spend a little bit of time talking about kind of just in the postpartum period, that sort of higher risk sometimes for certain patients for developing postpartum blues or depression and making sure that they and or their family or partner sort of knows some of those signs and knows to sort of seek help, giving people some resources off the bat for some of those things.*

[Provider 108]

One provider discussed how postpartum patients with OUD are at an increased risk for intimate partner violence (IPV). At patient visits, it can be important to assess for physical, emotional, and verbal abuse, and to provide resources and help if desired by the patient.

#### Theme 3: Acceptability of the Proposed Penny Chatbot

Clinicians agree that using a messaging system, such as Penny, for this population could be a positive addition to care. As this population has unique needs and requires increased care, this additional resource can be beneficial for a quick response to questions or support at virtually any time. Alternatively, outside of simple questions or resource distribution, more complex or sensitive matters may not be an ideal use of a messaging system. Issues shared with the chatbot should be able to be escalated promptly to the appropriate care provider, which may be challenging for this patient population as they often interact with multiple care clinicians or teams.

*I think that there is a lot of people involved in our patients’ care. They tend to have our team and then other medical providers across the hospital whether they may have- some of our patients, the baby is seen by us, but some go to a pediatrician and are followed by other groups and they're getting similar, if not the same information and uses them on who they need to go to for what. And then on top of that, there’s lots of social work interventions for the children and youth or shelters. We have a lot of folks in a lot of different systems and so a lot of professionals involved, so I would just worry that they wouldn't know like where the system is coming from and who they follow up to for what. At what point should be escalated, at what point should it be escalated to like a position or a direct outreach kind of?*

[Provider 107]

Clinicians discussed perceived barriers to engagement and the use of the Penny Chatbot among newly postpartum patients with OUD. As with general telehealth care, clinicians mentioned that a lack of stable phone access or reliable service may impact or prevent the use of Penny. Since the importance of establishing a personal connection with individual patients has been highlighted as necessary to promote adherence to treatment, clinicians expressed concern that a messaging system such as Penny may feel impersonal to this patient population. They also pointed out that information provided by the chatbot may not be clear and understandable to patients, which could make patients feel like they are not receiving the most accurate or best information and care. Clinicians pointed out that mistrust and privacy concerns may also be a barrier, as patients may not feel comfortable sharing certain information with a messaging system. However, issues of mistrust, privacy concerns, and comfort with using a chatbot were not identified as barriers by patients in any of the interviews conducted.

*I think that some people wouldn't utilize it because of, I think, the kind of mistrust and maybe not wanting to… say their business to an AI, or the internet, or whatever. I think that there are some issues in that. There might be some barriers to that. But I think that some people like the person who I just talked about… These questions about what’s okay, what’s safe in my pregnancy, what’s safe for the baby, I think that it would be really helpful for those kinds of questions for folks. I do think there is, yeah, I think that some people would be really hesitant to share some more like specifically around MOUD or use, just for privacy, or trust, or something like that.*

[Provider 102]

*And so sometimes when there’s discrepancies, even if they are potentially clinically insignificant discrepancies between what’s in the texting platform and what sort of a nurse or a doctor has said to them before. First of all, it can create confusion, but also it can create some mistrust of the whole situation and say, well, what is this automated thing? I want to talk to an actual person because I’m sort of confused by it and then I think even if the information is good, there can be some distance created when it’s like a computer that’s answering you instead of a doc.*

[Provider 103]

One clinician suggested that a way to combat mistrust about using Penny could be to introduce and explain the messaging platform to patients through a trusted or familiar source, such as a member of their care team.

*I think it would work best if a significant amount of trust in the system was built first. So it would need to be introduced by a trusted entity and also modeled and would work best if it was proven to be effective and helpful. I think that it could very quickly become a spam texting system if it’s not built out with the right amount of trust.*

[Provider 104]

One clinician described “text fatigue” as a potential barrier to engagement with Penny from this patient population, depending on how often Penny sends messages. Patients may already receive many text messages related to their care, so if Penny outputs an excessive number of messages, this may deter patients from engaging with the system.

*I do think that we have to be careful with all the texting that’s happening around oversaturation and overstimulation and alert fatigue, which I think is definitely a real thing now, much more than it was maybe in even like 2018, 2019, when some of our first text-based support platforms sort of became conceptualized. I know I get a lot more text messages now than I did five years ago. So, I think that it’s something we need to just be very cognizant of.*

[Provider 110]

When asked about potential advantages they may experience once Penny is implemented, most clinicians noted that a messaging system such as Penny could reduce workload and save time. Penny would answer most basic questions and concerns providers typically get while also providing patients with education and resource information. Clinicians would then be able to focus and spend more time with patients on concerns that may require specialized or a higher level of care.

Clinicians suggested that Penny interactions should be accessible and integrated within electronic health records (EHRs) so that providers can track care and focus on specific topics during visits. Another essential feature necessary for this chatbot to be implemented in this patient population is triage of care for more urgent or sensitive issues. Postpartum recovery, in addition to dealing with OUD, can make this patient population even more vulnerable, so implementation of depression or anxiety screenings through Penny can ensure an extra layer of care and support.

Patients (9/12, 75%) and providers (11/11, 100%) alike perceived the Penny Chatbot intervention to be useful. We defined perceived usefulness as the belief that the Penny Chatbot may be a beneficial addition to care for postpartum patients with OUD by quickly and conveniently answering questions, distributing vetted resources, and encouraging recovery maintenance. We assessed perceived usefulness in our patient and provider qualitative interviews.

## Discussion

### Principal Findings

This study reports on a qualitative needs assessment among perinatal women with OUD and the providers that care for them to determine what content would be most useful to include in an educational and supportive chatbot for those with perinatal OUD. Study participants universally reported that the Penny Chatbot may be a beneficial addition to care. Considering the unique and complex needs of a perinatal patient population with substance use disorders, providers suggest that Penny could promote recovery maintenance and engagement in treatment. Addiction care requires frequent check-ins. For a patient population that faces high treatment dropout and relapse rates along with barriers to in-person care, providers indicated that digital technology may be useful in increasing access to clinical support, bolstering provider-patient trust, and reducing after-hours call burden. Our findings are consistent with prior literature indicating that patients and providers alike agreed on the utility of digital means to connect patients to care [41,42]. Patients and clinicians agree that a balance of interactive chatbot content on substance use recovery, safe baby feeding practices, general postpartum and infant care, and connection to vetted resources would be helpful for postpartum mothers. Patients indicated that their greatest area of concern is understanding the impact of MOUD on their infant while breastfeeding. Patients also expressed strong interest in text-based support because of the availability, convenience, and instant responsiveness of a chatbot. Even so, they highlighted the importance of messaging that feels personal and sensitive to their needs. Given that the chatbot addresses key areas of concern and feels individualized to users, patients and clinicians believe that the Penny Chatbot may be acceptable and useful, with enthusiasm for the adapted intervention. As a result, our work will inform the development of supplemental material for the existing chatbot platform, specific to this patient population.

### Contribution to Literature

This study contributes to the existing literature highlighting the acceptability of mHealth to enhance clinical care for perinatal patients and those with substance use disorders. In samples of postpartum patients who do not use substances, chatbots have been found to be acceptable and usable for addressing postpartum mental health needs [23,24]. This mirrors our findings in that the majority of patients in this study reported that they would like and/or use a postpartum support chatbot.

Currently, the majority of digital health applications in the substance use field are devoted to substance use screening, telehealth visits, and virtual peer support [43]. Nonetheless, SMS messaging has been found to be feasible and acceptable as part of SUD treatment in addressing multiple dimensions of engagement, including motivation, self-efficacy, social support, and appointment attendance [44]. This study expands on perinatal OUD interventions, seeking to harness interactive, 2-way messaging to provide text-based clinical support to postpartum patients that may promote recovery, bolster patient-clinician trust, and help address loneliness.

This study also adds to the existing knowledge base on the value of participatory design. Through in-depth interviews with patients and healthcare providers who engage with perinatal OUD, we have obtained comprehensive and applicable qualitative feedback to inform the adaptation of a chatbot for postpartum OUD support.

### Needs Assessment Informed Penny Chatbot Development

To address the expressed interest in postpartum recovery information, we will maintain the existing content on vaginal delivery and cesarean section recovery (with planned individualization, as requested by patients) and modify the pain management recommendations to mirror the advice of perinatal addiction specialists. Content will include education on nonopioid pain management options, alternative therapies, monitoring for overuse or misuse, adjusting MOUD, and learning how to have open communication about pain management with providers.

There was ample support for education on infant withdrawal. Patients indicated they frequently use the internet to ask about what to expect and how to help their infant. Parenting messaging for all users with custody of their infant will include information on the “Eat, Sleep, Console” (ESC) protocol [45], Intensive Care Nursery (ICN) visiting information, signs of withdrawal, and tips for reducing or addressing withdrawal in newborns once discharged (swaddling, pacifiers, and minimizing smoke exposure).

The most common patient concern was safe infant feeding practices. Previous studies have pointed to discrepancies in breastfeeding among mothers using MOUD or abstaining and have highlighted the need for more support in this area for postpartum mothers with OUD [46]. Our findings suggest that patients want to know how substances affect their child’s development and want education on lactation and formula feeding while on MOUD. The chatbot will emphasize the safety of MOUD while breastfeeding [47]. We will also develop content on formula adjustment during infant withdrawal, what to do if a patient returns to use while breastfeeding, and the impact on infants of using different psychoactive substances while breastfeeding, including marijuana, nonprescribed benzodiazepines, and stimulants.

While mentioned by patients infrequently, providers indicated that mental health screening and support are critical for the patient population. To monitor risk for worsening perinatal anxiety and depression, the Edinburgh Postnatal Depression Scale (EPDS) [48] will be administered through the chatbot. Providers also noted high rates of IPV among perinatal individuals with OUD, so we will use the Extended-Hurt, Insulted, Threaten, Scream (E-HITS) [49] screening tool to monitor for IPV through the chatbot. Concerning scores will be escalated to the clinical team for direct outreach and patients will be texted resources.

Patients universally reported interest in increased postpartum OUD support. Information on how to secure a lactation consultation, free baby food and diapers, family support, infant soothing, and postpartum mental health resources provided by local public health and social organizations will be available through the chatbot. The chatbot will also have 24/7 functionality. Users will be able to ask questions at any time of day. By texting the word “Question,” patients can prompt a sequence of “if, then” responses to get the desired answers they seek. Questions that cannot be answered or require clinical attention, such as a request to be connected to a mental health therapist, will be streamlined to their care team.

Patients wanted messaging to feel individualized and personal. Chatbot messaging will be developed to sound warm and encouraging, as if the patient is texting a friend. Patients will receive a warm greeting with the first message of each day, as well as a thoughtful and brief introduction before the delivery of sensitive content related to substance use. Providers warned that too many messages may overwhelm patients. Messaging will be limited to no more than 3 times a day to minimize the burden of message fatigue, and messages will decrease in frequency as the 6-week protocol goes on. To enhance personalization, the chatbot will use the patient’s name, infant’s name, and provide their provider’s phone number. We will also opt users in and out of text message content depending on what information corresponds with their birth and postpartum experience. Patients will receive either cesarean or vaginal delivery recovery content and lactation or formula feeding content. Patients who do not go home with their infant due to the child’s extended neonatal intensive care unit (NICU) stay or family services involvement will not receive information related to daily pediatric care.

### Acceptability of the Proposed Penny Chatbot

The Penny Chatbot was described as a potentially beneficial supplemental support to postpartum individuals with OUD. As with other digital interventions for people with OUD, our data suggest that an engaging, sensitive, and individualized chatbot has value in increasing access to education, recovery maintenance, and vetted resources [50]. Providers warned that the chatbot’s effectiveness may be impacted by patient distrust, escalation protocol, and text fatigue. However, we will address these concerns by introducing the chatbot through a warm hand-off by a trusted clinician and giving a detailed explanation of aspects of the platform, such as its privacy and confidentiality. Patients will be made aware that a member of the care team will be notified and will follow up with them in the event of certain concerning responses, such as with mental health or intimate partner violence screening measures. Finally, we will ensure that patients are only receiving relevant and desired messaging. Users will be able to unenroll and stop messaging at any time.

### Strengths

This needs assessment demonstrated the application and usefulness of participatory design. Involving perinatal patients with OUD and clinicians who engage in addiction treatment, perinatal care, or a combination of both as stakeholders in the research process ensures that the adaptation of the Penny Chatbot will effectively address user needs, preferences, and concerns [51]. This needs assessment directly guides text-based content development, resource selection, screening measures, and overall communication style: frequency, wording, and tone.

### Limitations

We acknowledge that there are limitations in this study. The patient sample consisted of patients from a single perinatal OUD treatment center at the University of Pennsylvania, limiting the generalizability of the data to all perinatal patients with OUD. Further, all clinicians who were interviewed were employees of the same health system, limiting the generalizability of study findings.

### Conclusion

Postpartum chatbots offer an interactive experience, providing 24/7 clinical answers, maternal mental health resources, and embedded intervention [34]. Text message–based delivery is ubiquitous and adaptive to the needs of an immediate postpartum patient population. mHealth technology access is not limited to higher-income populations; thus, chatbots can address barriers associated with stigma, affordability, and limited access to in-person medical care [52,53]. Chatbots have the potential to increase access to evidence-based mental health and substance abuse care for underserved groups [21]. As barriers to OUD care, especially during the perinatal period, include clinic access factors and stigma, we believe that the development of a postpartum OUD chatbot program using the information described herein addresses a gap in the existing knowledge base on mHealth for perinatal SUD as a way to provide a cost-effective and easily disseminated tool to improve OUD treatment outcomes [54].

Considering this, our team will use these data to further refine and test the Penny Chatbot. Future research on postpartum OUD chatbots should further explore and expand on content areas most useful and desired by this population, as well as ways clinical teams can manage ongoing monitoring to address urgent needs in a responsive manner. An in-depth participatory-driven needs assessment allows for the adaptation of the existing chatbot to meet the needs of a high-risk, difficult-to-reach population. As we move forward with testing of the chatbot for acceptability and feasibility, data from the needs assessment allow us to iteratively refine its content, delivery, and safety.

## Abbreviations

COREQ: Consolidated Criteria for Reporting Qualitative Research
CRS: certified recovery specialist
E-HITS: Extended-Hurt, Insulted, Threaten, Scream
EHR: electronic health record
EPDS: Edinburgh Postnatal Depression Scale
ESC: Eat, Sleep, Console
HIPAA: Health Insurance Portability and Accountability Act
ICN: Intensive Care Nursery
IPV: intimate partner violence
IRB: institutional review board
IRR: interrater reliability
mHealth: mobile health
MMRL: Mixed Methods Research Lab
MOUD: medications for opioid use disorder
NICU: neonatal intensive care unit
NOWS: Neonatal Opioid Withdrawal Syndrome
OUD: opioid use disorder
PROUD: Perinatal Resources for Opioid Use Disorder
